# Effects of an intervention program with health education and hatha yoga on the health of professionals with musculoskeletal symptoms

**DOI:** 10.47626/1679-4435-2020-492

**Published:** 2020-12-11

**Authors:** Fernanda Mazzoni da Costa, Nelson Filice de Barros, Henrique Ceretta de Oliveira, Neusa Maria Costa Alexandre

**Affiliations:** 1 Doutorado Interinstitucional, Universidade Estadual de Campinas/Universidade Federal de Juiz de Fora – Campinas (SP), Brazil.; 2 Faculdade de Ciências Médicas, Universidade Estadual de Campinas - Campinas (SP), Brazil; 3 Faculdade de Enfermagem, Universidade Estadual de Campinas - Campinas (SP), Brazil

**Keywords:** occupational health, musculoskeletal pain, psychological stress, yoga, health education

## Abstract

**Introduction:**

Musculoskeletal and mental disorders are relevant in the workers’ disease process, and ergonomic interventions that include guidance and physical exercise consist of strategies of health promotion. Integrative and complementary practices are presented as a possibility of promoting comprehensive care and yoga consists of a therapeutic alternative.

**Objective:**

To evaluate the effects of an intervention including educational measures and hatha yoga in musculoskeletal pain, disability, and stress in professionals of a university hospital.

**Methods:**

We selected 125 professionals with musculoskeletal symptoms of intensity ≥ 1 who did not practice yoga and randomly assigned them to intervention (n = 63) and control (n = 62) groups, requesting answers to the following questionnaires: initial characterization, the Nordic Musculoskeletal Questionnaire and a numeric scale, the Pain Disability Questionnaire, and the Perceived Stress Scale. The intervention group went through a 12-week program with educational measures and hatha yoga. At the end of the study period, both groups answered to the questionnaires once again. We compared data before and after the intervention and between groups.

**Results:**

Both groups presented improvements after 12 weeks, but the difference between mean results obtained in the first and second data collections revealed that the levels of pain, disability, and stress decreased more strongly in the intervention group than in the control group. Considering that the intervention group began the program in worse clinical conditions, the program led to a reduction in the difference between groups, but this was not enough for the intervention group to reach better results than the control.

**Conclusions:**

The intervention promoted improvements in the intensity of pain, disability, and stress among the participants of the intervention group. Similar programs could be explored in the promotion of occupational health.

## INTRODUCTION

The current world of labor presents demands that often exceed the natural limits of the workers’ skills and capacities, leading to physical, psychological, and social dysfunctions that affect health, social relations, and productivity. A study by the Brazilian Ministry of Labor, Employment, and Social Security on disability benefits has shown the relevance of musculoskeletal and mental disorders in the disease process of workers in Brazil.^1^

Ergonomic interventions can be used as a strategy for promoting health. Studies have suggested the combination of various resources in the same program,^2^ and researchers have investigated the efficacy of educational activities^3^^,^^4^ and exercises^2^^,^^5^^-^^9^ in musculoskeletal symptoms, the related disability, and stress.

Actions for disease prevention and for the promotion, maintenance, and restoration of health can be developed from various sources of knowledge and practices. A significant and increasing part of the global population, frequently underestimated by health care systems and services, turns to unconventional practices as a basis or complementary aspect of strategies for promoting health and preventing or treating mainly chronic diseases.^10^^-^^12^ This reveals that the western scientific health care model is not the only pattern of care and some therapeutic systems are guided by other health paradigms.^13^ Therapies based on bioenergetics stimulate self-care, contribute in modulating the immune response, and induce lifestyle changes, thus providing alternatives to the limits of the hegemonic medical model.^10^

In order to promote the safe and effective use of other therapeutic systems, the World Health Organization (WHO) aims to support the member states through the Traditional Medicine Strategy 20142023.^11^ In Brazil, aiming at a comprehensive health care and the principles of the Unified Health System (SUS), the National Policy for Integrative and Complementary Practices (PNPIC)^14^^,^^15^ was instituted for officiating non-hegemonic practices within the SUS. The National Policies for Humanization (PNH), Primary Health Care (PNAB), Health Promotion (PNPS), and Popular Health Education (PNEPS) are also of note.

Yoga is among the WHO-recognized integrative and complementary practices (PIC) listed in the SUS table of procedures, medications, ortheses, prostheses, and special materials for primary care and the Academia da Saúde program. Originally from India, and initially transmitted from masters to apprentices, yoga was structured by Patañjali in the classic text Yoga Sutra, which describes an 8-part system (ashtanga) consisting of an ethicophilosophical subject comprising abstinences (yama) and observances (niyama), postures (asanas), control of the vital energy (pranayama), of sensations (pratyahara), concentration (dharana), meditation (dhyana), and control of the mind (samadhi). This practice contributes to reducing mental agitation and promotes the necessary conditions for self-knowledge and for perceiving one’s connection with the creator energy.^16^

Although the philosophical system of yoga is not limited to a form of treatment, studies have shown that its practice can contribute to the promotion of health by providing physical, philosophical, and social benefits,^1^ and in the treatment of people with musculoskeletal and mental disorders.^18^ However, research on yoga is incipient and the available studies have limitations.^19^ Moreover, not all people achieve benefits through yoga, although it is a valid treatment option for most of its practitioners.^17^

In this context, the present study aimed to analyze the effects of an intervention program comprising health education actions and hatha yoga on the intensity of musculoskeletal pain, the related disability, and stress among workers of a university hospital.

## METHODS

This is an intervention study performed with professionals in the assistance, support, and administrative sectors of the outpatient unit of a university hospital in the state of Minas Gerais, with no occupation restriction; the total population included 232 people, of which 174 accepted to participate in our study. Research assistants distributed and collected questionnaires among participants. The inclusion criterion was the occurrence of a musculoskeletal symptom in any body region in the last 12 months, which was verified by the Brazilian version of the Nordic Musculoskeletal Questionnaire^12^; the region with the most intense pain was considered in the analysis. Out of 165 received questionnaires, 127 participants reported symptoms. Exclusion criteria were pain with intensity lower than 1 and participants who practiced yoga at least once a week. These participants were identified through a numeric scale^21^ and a question regarding regular yoga practices. We identified 125 professionals presenting musculoskeletal symptoms with intensity ≥ 1 and who did not practice yoga. These participants were randomly distributed in intervention (IG, n = 63) and control groups (CG, n = 62).

Both groups answered a sociodemographic and occupational questionnaire adapted from the National Demographic Census (2010); the Nordic Musculoskeletal Questionnaire and a numeric scale; the Brazilian version of the Pain Disability Questionnaire (including an evaluation of functional, psychosocial, and total disability)^22^; and the Perceived Stress Scale.^23^

Professionals allocated in the IG were invited to participate in a 12-week intervention program approaching health education and hatha yoga sessions. In the following weeks, these participants were reminded of the program by posters, WhatsApp messages, and monthly letters.

Within the health education activity, we organized a group discussion about ergonomics and posture at work and distributed informative material that synthesized the approached subject. The hatha yoga sessions, based in the classic Patañjali yoga, included 15 minutes of a theoretical approach in the first session in order to clarify the objectives and methods and the distribution of informative material. Weekly group yoga sessions lasted 60 minutes for 12 consecutive weeks. The yoga sessions included exercises for the joints, breathing, strength, stretching, relaxation, and concentration, indicated for all participants but adapted to each case. The activities took place in an appropriate setting inside the hospital and each session was repeated 4 times per week in different days and hours, when work demands were low, in order to facilitate participation. We did not perform any intervention with the CG.

At the end of the program, participants of both groups answered to the Nordic, Pain Disability, and Perceived Stress questionnaires. Data collection happened from August 2016 to January 2017.

We used the Statistical Analysis System (SAS) software, version 9.4, for data analysis. Variables considering the sociographic characterization, occupational aspects, and pain intensity for each group were described through position and dispersion measures in the case of quantitative variables and through frequencies and percentages when considering qualitative variables. Quantitative variables were evaluated between the 2 groups using an unpaired Student’s t-test or a Mann-Whitney test, according to data distribution. The Shapiro-Wilk test was used for verifying whether data followed a normal distribution. Qualitative variables were evaluated through a Fisher’s exact test.

Pain intensity and the related disability, as well as the perceived stress, were measured in the 2 moments of evaluation (before and after the 12-week program). In order to compare the groups and moments with regards to pain-related disability and perceived stress, we used linear mixed-effects models and applied the Box-Cox transformation to dependent variables.

For comparing pain intensity results, we used the Mann-Whitney test and Wilcoxon paired test with a Bonferroni correction considering a significance level of 1.25%. We selected these analyses instead of mixed models due to the fact that pain intensity was a discrete quantitative variable.

This study was authorized by the hospital and approved by the Research Ethics Committee (decision No. 1 280 516, Certificate of Presentation for Ethical Consideration [CAAE] 46094315 4 0000 5133). Participation in this study was voluntary and subjected to the signing of a free and informed consent form.

## RESULTS

**Figure 1:**
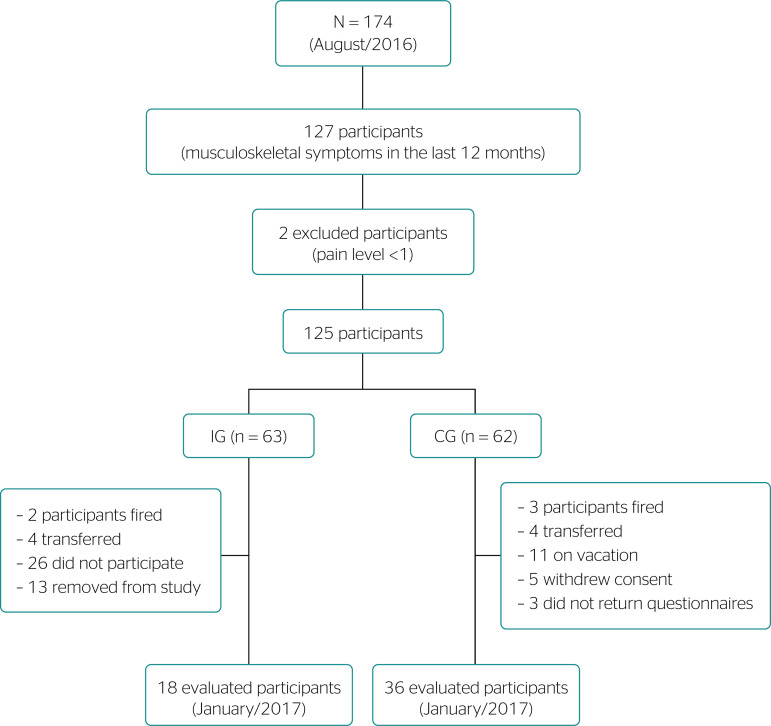
Participant selection flowchart. Juiz de Fora, 2017 (N = 54).

Within the IG, 2 participants were fired, 4 were transferred, 26 did not participate in the hatha yoga sessions, and 13 were removed from the study due to insufficient attendance (less than 4 sessions); altogether, we obtained complete data for 18 participants. In the CG, 3 participants were fired, 4 were transferred, 11 were on vacation, 5 withdrew consent, and 3 did not return our questionnaires, hence this group totaled 36 participants (Figure 1).

The groups did not present a statistically significant difference in age (p = 0.92) and both contained young participants (in the CG, mean age was 38.67 and standard deviation [SD] was 10.22; in the IG, mean age was 38.94 and SD was 11.49). The groups did not present differences regarding weekly working hours (p = 0.11), whose mean values were 40 in the CG and 42 in the IG; interquartile intervals were 4 and 24, respectively (Table 1).

**Table 1:** Characterization of participants as to age and weekly working hours. Juiz de Fora, 2017 (N = 54).

Variable	Group	Mean	Standard deviation	Minimum	First quartile	Median	Third quartile	Maximum	p
Age (years)	Control (n = 36)	38.67	10.22	22	30	36	46.5	60	0.92*
	Intervention (n = 18)	38.94	11.49	24	27	37	48	57	
Weekly working hours	Control (n = 36)	39.11	5.56	30	36	40	40	56	cm^†^
	Intervention (n = 18)	45.11	12.08	30	36	42	60	72	

* p obtained with an unpaired Student's t-test;

† p obtained with a Mann-Whitney test.

Most participants were female (GC = 66.67%; IG = 100%, p = 0.00), had an undergraduate degree (CG = 58.33%; IG = 66.67%, p = 0.89), had only one job (CG = 94.44%; IG = 83.33%, p = 0.31), was privately employed by the hospital (CG = 69.44%; IG = 66.66%, p = 0.03), and worked in assistance sectors (CG = 44.44%; IG = 83.33%, p = 0.02) (Table 2).

**Table 2:** Characterization of participants as to sex, education, number of jobs, type of employment, and sector. Juiz de Fora, 2017 (N = 54).

Variable	Group				p*
	Control		Intervention		
	n	%	n	%	
Sex					0.00
Male	12	33.33	-	-	
Female	24	66.67	18	100	
Total	36	100	18	100	
Education					0.89
Secondary education	15	41.67	6	33.33	
Undergraduate education	9	25	5	27.78	
Graduate education (specialization)	9	25	6	33.33	
Graduate education (master's)	3	8.33	1	5.56	
Total	36	100	18	100	
Number of jobs					0.31
1	34	94.44	15	83.33	
2 or more	2	5.56	3	16.67	
Total	36	100	18	100	
Form of employment					0.03
Privately employed	25	69.44	12	66.66	
Employed by the public sector	11	30.56	3	16.67	
Resident	-	-	3	16.67	
Total	36	100	18	100	
Sector					0.02
Administrative	6	16.67	1	5.56	
Support	14	38.89	2	11.11	
Assistance	16	44.44	15	83.33	
Total	36	100	18	100	

* p obtained with a Fisher's exact test.

The lower back was the body region with the most occurrences of pain of highest intensity in both groups (CG = 33.33%; IG = 55.55%) (Table 3).

**Table 3:** Body regions with pain of highest intensity, per group. Juiz de Fora, 2017 (N = 54).

Body region with pain of highest intensity	Group			
	Control		Intervention	
	n	%	n	%
Lower back	12	33.33	10	55.55
Ankles/feet	6	16.66	2	11.10
Neck	4	11.11	3	16.67
Shoulders	4	11.11	-	-
Knees	4	11.11	1	5.56
Upper back	2	5.56	1	5.56
Wrists/hands	2	5.56	1	5.56
Hip/thighs	2	5.56	-	-
Total	36	100	18	100

Before the intervention, the IG presented, in comparison to the CG, higher mean scores of pain intensity (CG = 5.64; IG = 7.17, p = 0.06), functional (CG = 14.25; IG = 25.06, p = 0.00), psychosocial (CG = 8.94; IG = 16.17, p = 0.01), and total disability (CG = 23.19; IG = 41,22, p = 0.00), as well as stress (CG = 17.58; IG = 18.89, p = 0.39); differences were statistically significant for functional, psychosocial, and total disability. After the intervention program, both groups exhibited lower levels of pain intensity (CG = 2.72, p < 0.0001; IG = 2.89, p = 0.0006), functional (CG = 13.19, p = 0.28; IG = 21.61, p = 0.17), psychosocial (CG = 8.31, p = 0.42; IG = 12.22, p = 0.17), and total disability (CG = 21.50, p = 0.27; IG = 33.83, p = 0.10), and stress (CG = 16.47, p = 0.22; IG = 16.89, p = 0.13). The difference was statistically significant for pain levels.

However, the differences between mean results observed before and after intervention in each group revealed that the IG had a stronger reduction in pain intensity (CG = 2.92; IG = 4.28. p = 0.63), functional (CG = 1.06; IG = 3.12, p = 0.02), psychosocial (CG = 0.63; IG = 3.95, p = 0.07), and total disability (CG = 1.69; IG = 7.39, p = 0.03), and stress (CG = 1.11; IG = 2.00, p = 0.77). Statistically significant differences were observed in functional and total disability. Table 4 compares pain intensity, the related disability, and stress before and after the intervention program.

**Table 4:** Comparison of pain intensity, pain-related disability, and stress before and after the intervention program. Juiz de Fora, 2017 (N = 54).

Variable	Moment	Intervention Group (n = 18)		Control Group (n = 36)		p (groups)
		Mean (SD)	p (moments)	Mean (SD)	p (moments)	
Pain intensity	Before	7.17 (3.01)	0.00*	5.64 (2.43)	<0.00*	0.06^†^
	After	2.89 (2.65)		2.72 (3.07)		0.63^†^
Functional disability^‡^	Before	25.06 (17.12)	0.17	14.25 (8.11)	0.28	0.00
	After	21.61 (13.84)		13.19 (7.31)		0.02
Psychosocial disability^‡^	Before	16.17 (13.51)	0.17	8.94 (5.95)	0.42	0.01
	After	12.22 (8.34)		8.31 (4.35)		0.07
Total disability^‡^	Before	41.22 (30.03)	0.10	23.19 (12.57)	0.27	0.00
	After	33.83 (21.52)		21.50 (11.18)		0.03
Perceived stress ^‡^	Before	18.89 (5.88)	0.13	17.58 (4.72)	0.22	0.39
	After	16.89 (5.14)		16.47 (5.06)		0.77

SD: standard deviation.

* p obtained with a Wilcoxon paired test;

† p obtained with a Mann-Whitney test;

‡ p obtained with linear mixed-effects models.

## DISCUSSION

Despite our strategies for promoting program attendance, we observed an expressive loss of participants in both groups, which could have been influenced by high workloads, difficulties in finding free time during working hours, strikes, and vacations. Attendance can also be evaluated considering that this is a treatment that proposes lifestyle changes and requires active participation, as opposed to conventional treatments that can be received in a passive manner. Low attendance is the main limitation described by similar studies^24^^-^^26^ and can influence results, since behavioral interventions rely on active participation.^26^

This study has limitations that should be noted. In order to obtain a sufficient number of participants in a limited population and with the adoption of inclusion and exclusion criteria, we included all individuals with musculoskeletal symptoms, regardless of their duration. The random differences in group compositions resulted in a possible sociodemographic confounding bias. The number of selected individuals and the program attendance rates were low, which compromised our results in matter of statistically significant differences and precluded our comparisons; hence results of one individual may have affected mean values. External factors, such as medications and other treatments, were not controlled. Lifestyle changes also require a longer period than that of our study to be consolidated.

Most of our sample consisted of young professionals, around midlife economically active, female, with undergraduate education, who worked weekly hours according to the Brazilian law, had only one job, were privately employed by the hospital (CLT regimen) in assistance sectors, and had most intense pain in the lower back. Despite a random distribution, we had different pre-intervention results between groups: the IG presented higher levels of pain, disability, and stress.

The mean results obtained after the intervention showed that both groups presented improvements with time, although the only statistically significant result was the one for pain levels. When comparing differences (before and after intervention) within both groups, we noted that pain, disability, and stress had a bigger reduction in the IG than in the CG, which highlighted positive effects of the intervention on all analyzed variables even though functional and total disability were the only ones to have reached statistically significant differences.

Nevertheless, considering that professionals from the IG began the study in worse clinical conditions when compared to the CG, after the intervention this difference between groups was reduced, but not enough for the levels of pain, disability, and stress in the IG to become lower than those in the CG. However, the results suggest that the intervention, if continued, could promote improvements for the IG.

Health promotion programs should perform individual and collective actions aimed at improving the work environment and processes, as well as psychosocial demands and lifestyle (mainly considering physical activity, musculoskeletal conditioning, and a reduction in body mass).^2^ Our program did not interfere in work organization conditions, but sought to provide actions (health education and hatha yoga) that could influence the performance of work processes, psychosocial aspects, and promote lifestyle changes, in addition to encouraging physical activity.

Our 12-week program included a health education activity that sought to provide tools for identifying factors that contributed to musculoskeletal disorders, for comprehending the individual and organizational responsibilities, as well as adopting safe attitudes at work, social interaction strategies, and pain coping. Moreover, the hatha yoga sessions included postures that demanded muscle strength and endurance, stretching, and relaxation, in addition to exercises for the joints, breathing, and concentration; the sessions were performed at the workplace and during working hours under the supervision of a qualified instructor. Altogether, our results showed that the program reduced pain intensity, the related disability, and stress among participants.

The efficacy of educational activities in the management of musculoskeletal pain has been investigated.^3^^,^^4^^,^^27^^,^^28^ Relevant guidelines recommend guidance and physical activity, but there is no consensus as to the type of activity. Studies have demonstrated the efficacy of programs that involve muscle strength and endurance exercises, as well as stretching and relaxation, in preventing injuries and reducing musculoskeletal symptoms.^2^^,^^6^^,^^7^ Programs that include supervised resistance exercises for periods longer than 10 weeks presented strong evidence in the control of neck pain in office workers, and moderate evidence for low-back pain in workers that perform manual handling of loads.^5^ Moreover, programs that are performed at the worksite have been shown to be more effective than home-based ones.^8^

Research on the therapeutic use of yoga has been performed throughout the last 4 decades, although in relatively low numbers; most studies are small, with low participation and high participant loss rates, of low quality, and very heterogeneous, which hinders the comprehension of the benefits of this activity. Although the potential of yoga as a healing treatment has not yet been defined, this activity contributes as a behavioral self-care intervention in complementary treatments by promoting lifestyle changes.^26^ Occupational health interventions using yoga have provided results in health promotion, pain reduction, the development of psychosocial skills, quality of life, self-care, and work performance; however, these have been published by a small number of heterogeneous studies with small samples, muddling the conclusion process. Although there are indications that yoga is related to positive physical and psychological effects in workers, more research is necessary for elaborating this conclusion.^19^

Some studies have demonstrated the efficacy of physical exercise in workers’ musculoskeletal symptoms, but not in psychosocial aspects such as stress.^7^^,^^9^ Yoga has been shown to be useful in musculoskeletal pain because it increases flexibility, demonstrates to patients that they can remain active despite the pain, eases the mind and helps with concentration, increases consciousness, decreases anxiety and anguish, and improves the mood. Regarding stress, yoga was demonstrated to be as effective as relaxation, cognitive-behavioral therapy, or dancing,^26^ which indicates that its effects are not limited to those provided by the physical activity.^16^

The mindfulness practice should be highlighted as an important aspect for the comprehension of the effects of yoga because it could be the component that differentiates this activity from other modalities of physical exercise. In this process, beyond the physical exercise and its associated benefits, each action is performed slowly and maintained for a determined time with a focus on breathing and consciousness of the physical and mental experience of the moment before moving on to the next action; this includes a mental component that favors full attention throughout the activity.^24^ Various meditation methods exercise present moment awareness aiming to change the manner with which an individual relates to random thoughts that constantly travel between the past and the future, agitating the mind; this is named rumination.^29^ Studies indicate that mindfulness-based activities promote physical and psychological benefits,^24^^,^^29^^-^^34^ with evidence of efficacy on pain intensity and stress^24^^,^^34^; results also suggest a potential for helping individuals to learn how to handle their clinical and nonclinical problems, thus promoting physical and psychological wellbeing.^33^

The effects observed in this study may be related to the mindfulness component of the hatha yoga sessions. Concentrating in the present moment promotes calming effects in the sympathetic nervous system that generate physiological changes (a reduction in respiratory and cardiac frequencies, of oxygen consumption, arterial pressure, muscle tension, and a change in brain waves) and contribute to reducing rumination, changing negative thought patterns.^29^^,^^32^^,^^35^^,^^36^ The time invested in meditation activities such as yoga is associated to mindfulness, health, and wellbeing,^30^ and advanced yoga practitioners exhibit significantly higher levels of mindfulness and lower levels of stress.^29^ This ability can be developed through training,^30^^,^^31^ and many participants of interventions such as the one performed in this study continue practicing the activity after the end of the program.^24^

This study reported an approach within the concept of health education and yoga that contributed to the construction of care modes founded in an individualized and comprehensive perspective. It described results regarding pain, disability, and stress, and evaluated behavioral and psychosocial components that provide tools for an individual to rethink his or her way of living and working and to handle physical and psychological suffering; our approach also allowed the development of coping and self-management skills for possible physical, psychological, and social problems.

Considering the possibilities emerging from the implemented program, we highlight that although not all variables showed statistically significant differences, our findings suggest that the most evident effects of the yoga sessions could continue contributing with behavioral changes and their effects on the health and wellbeing of participants in the long term. However, the current scientific research model presents challenges for designing studies on this subject, since PIC have an individualized approach and their results rely on the conscious choice and active engagement by the individual in his or her healing process.^37^^,^^38^

Moreover, our program showed that complex interventions have a set of therapeutic elements that act simultaneously and synergistically.^12^^,^^38^ In addition to the expected results, treatments frequently promote nonspecific effects that may include effects of the observation and evaluation, the administration of a mock treatment, and the interaction between the therapist and the patient.^37^ These are context effects that derive from the meaning of the intervention for the individual, and are influenced by his or her relationship with the therapist, the environment, as well as by the patient’s beliefs, with a strong and clinically important effect that should not be ignored; these effects also provide information on how the body, the mind, and the culture influence self-healing results.^39^^,^^40^ Specific and nonspecific effects are synergistic and it is not possible nor desirable to separate them. Considering only results that can be attributed to a particular component of the intervention is incurring in the efficacy paradox.^12^^,^^38^^,^^39^^,^^40^

The PIC promote a global effect that is bigger than the sum of its parts, and the lack of efficacy in one component should not be used for discrediting the result.^12^ Clinical decisions consider, in addition to research results, factors related to professionals/care providers and patient preferences^38^. Medicine grounded only on scientific competencies is insufficient in finding meaning in the disease process and coping with health loss. A care model focused on the person recognizes the need for adding to scientific evidence methods that are capable of evaluating the human being in all its dimensions. We should consider the facts that influence clinical decision making^38^ and think of different and more complex research strategies for PIC.^12^

## CONCLUSIONS

Our results showed that the program promoted improvements in pain intensity, disability, and stress among participants of the IG and indicated that similar interventions could be an alternative for promoting occupational health, considering the possibilities and challenges in the implementation of PIC and the limits and perspectives of research on this subject.

Mean pain intensity, disability, and stress scores after intervention demonstrated that both groups had improvements in all variables, even though pain intensity was the only variable that demonstrated a statistically significant difference. However, when comparing the differences between mean results of both groups before and after the intervention, we observed that pain, disability, and stress decreased more intensely in the IG than in the CG, revealing positive effects of the intervention on all analyzed variables, although only the functional and total disability scores reached statistically significant differences.

Considering that the IG had a worse initial clinical condition when compared to the CG, our results indicated a reduction in this difference between groups, but not enough for the pain, disability, and stress levels after intervention to become better in the IG; nevertheless, our results suggest that a continued intervention should promote advantages for the IG. Therefore, we suggest that new studies on the development of programs comprising different health-promoting activities should be performed.
